# Daily Intake Estimation for Young Children’s Ingestion of Residential Dust and Soils Contaminated with Chlorpyrifos and Cypermethrin in Taiwan

**DOI:** 10.3390/ijerph15071327

**Published:** 2018-06-25

**Authors:** Ya-Qing Yang, Lih-Ming Yiin

**Affiliations:** Department of Public Health, Tzu Chi University, 701, Sec. 3, Zhongyang Road, Hualien City 97004, Taiwan; sunnysunnyireen@gmail.com

**Keywords:** chlorpyrifos, cypermethrin, daily intake, home environment, Monte Carlo simulation, non-dietary ingestion, SHEDS model, Taiwan

## Abstract

We estimated the daily intakes of chlorpyrifos and cypermethrin via ingestion of indoor dust and outdoor soils using the Stochastic Human Exposure and Dose Simulation Model on a probabilistic approach for Taiwanese young children. Variables for the estimation, such as concentration, ingestion rate, and body weight, were adopted from previous studies. Monte Carlo simulation was performed with 1,000,000 iterations to simulate a single daily intake, which was shown in terms of percentage of the Acceptable Daily Intake (ADI) of either insecticide. The daily intakes are minimal with a 99% probability, but go up steeply at the 99.9th percentile (13.1% and 20.0% of the ADIs of chlorpyrifos and cypermethrin, respectively). The sensitivity analysis indicates that concentration is the most determinant variable for daily intake estimation, suggesting that high intakes may occur when insecticide concentrations are elevated. Compared to the data of daily intakes via dietary ingestion of vegetables derived from a previous study, the estimated non-dietary intakes are negligible until reaching the highest percentile. Consequently, the non-dietary ingestion exposure to either insecticide is commonly low for young children in Taiwan’s homes, unless high contamination (e.g., indoor insecticide application) occurs in the environment. Care has to be taken to avoid high contamination indoors.

## 1. Introduction

Taiwan is a perennially warm and humid island, where pesticides are frequently used for agriculture and vector-borne disease control. A previous report indicated that Taiwan was among the top countries around the world in pesticide consumption [1]. With such substantial consumption in quantity, residues of insecticides present in residential environments are nearly inevitable. A pilot study conducted in Taiwan has demonstrated high detection rates of chlorpyrifos and cypermethrin in house dust, suggesting insecticide use in homes [2]. Residues of insecticides in the home environment serve as a potential health threat to young children, because their hand-to-mouth behaviors may enhance the non-dietary ingestion exposure to insecticides [3].

Chlorpyrifos and cypermethrin are different types of insecticides (i.e., organophosphate, pyrethroid), but both serve as neurotoxic agents to kill bugs. A number of studies have indicated that prenatal exposure to organophosphate and/or pyrethroid insecticides is associated with children’s behavioral disorders or neurodevelopmental problems [4,5,6,7,8,9,10,11,12,13,14]; moreover, recent studies have found that postnatal childhood exposure to insecticides may have similar effects [15,16,17,18], suggesting young children’s susceptibility to insecticides. Childhood exposure may occur via dietary ingestion, such as consumption of fruits and vegetables contaminated with insecticides, and via non-dietary ingestion (e.g., by mouthing behaviors), which is of great concern should the environment be highly contaminated. A previous study, conducting an exposure assessment of chlorpyrifos on residential surfaces for young children at 3–6 years old one week after an indoor application, has found the dose via non-dietary ingestion to be 126 µg/kg/day [3]. Another study using a probabilistic modeling framework to estimate children’s non-dietary ingestion of chlorpyrifos has indicated the daily ingested chlorpyrifos being approximately 1000 µg/day (median value) and slightly lower than 100 µg/day (median value) within and after the first week of application, respectively [19]. These estimates that show high doses few days after application suggest that non-dietary ingestion could be an important route of exposure to insecticides for children in the residential environments.

The modeling used in the previous study [19] is the Stochastic Human Exposure and Dose Simulation Model (SHEDS) for multiple pollutants, which is a probabilistic human-activity-based physical model developed by the U.S. Environmental Protection Agency (EPA) [20]. It is designed to estimate dust and soil ingestion exposure to pollutants, with application of videographic data of children’s everyday activities indoors and outdoors to indicate frequencies and types of contact with various surfaces, instead of using tracer-element-based mass balance models [21]. In addition, this strategy of modeling predicts full variability distributions of dust and soil ingestion rates for age-specific groups, which are better than traditional single-point estimates. Coupled with Monte Carlo simulation, a method using repeated random sampling to generate simulated data, SHEDS could provide a decent estimate of non-dietary dust and soil ingestion exposure to insecticides for young children in the home environment, with variability and uncertainty being considered.

Since Taiwan uses large quantities of pesticides every year, it is interesting to know residential exposure to insecticide residues particularly for young children. The previous studies that estimated non-dietary ingestion exposure to insecticides were all conducted short after application, mimicking worst-case scenarios. This study intended to assess the exposure on a regular basis, and to include variability and uncertainty for the outcome. Thus, we introduced the concentration profiles of chlorpyrifos and cypermethrin previously measured from residential environments by our research team [2] and the dust and soil ingestion rates for 3–6 year old children estimated by SHEDS [21] to Monte Carlo simulation to predict the non-dietary ingestion exposure to chlorpyrifos and cypermethrin for young children in the home environments of Taiwan. To our best knowledge, this is the first study that estimates residential exposure via non-dietary ingestion of insecticides using a probabilistic approach for children in Taiwan.

## 2. Materials and Methods

### 2.1. Analysis of Insecticides in Dust or Soils

Sampling of house dust and yard soils was conducted in participating homes with consent. The study was conducted in compliance with the Declaration of Helsinki, and the protocol and procedures were reviewed and approved by the Ethical Committee of Tzu Chi General Hospital/University (No: IRB103-170-B, approved on 5 January 2016). A composite sample of indoor dust and an outdoor soil sample were collected from each house using a vacuum sampler and a set of dustpan/brush set, respectively. Approximately 0.5 g of dust or soil from each sample was treated with ethyl acetate for ultrasonic extraction and centrifugation, and the extract was analyzed on gas chromatography-mass spectrometry for chlorpyrifos and cypermethrin with use of selective ion monitoring for sensitivity enhancement. The limits of detection (LOD) were found to be 0.025 and 0.031 µg/g for chlorpyrifos and cypermethrin, respectively. Replicate analysis was conducted for randomly selected dust and soil samples for variability check, which showed good reproducibility. There were 52 indoor and 57 outdoor samples with valid data of chlorpyrifos and cypermethrin for the study. Details of sampling and analytic procedures were described previously [2].

### 2.2. Estimation of Daily Intake

The formula for daily intake via ingestion of dust or soils is as follows:

(1) $$\mathrm{Daily}\;\mathrm{intake}=\frac{\mathrm{IR}\times C\times \mathrm{CF}}{\mathrm{BW}}$$

where

IR: Ingestion rate of dust or soils (mg/day)

C: Concentration of insecticide in dust or soils (chlorpyrifos or cypermethrin) (µg/g)

CF: Conversion factor (10^−3^ g/mg)

BW: Body weight of children (kg)

Distributions of the above variables are listed in Table 1. Ingestion rates of dust or soils adopted herein were modeled specifically for 3–6 year old children using SHEDS, because of high frequency of mouthing behaviors and availability of adequate exposure data for this age group [21]. Concentrations of insecticides (chlorpyrifos, cypermethrin) for indoor dust and outdoor soils, provided by our previous work [2], could be used for calculation with ingestion rates of dust and soils, respectively. For those dust samples under LOD, halves of the LOD values were used in place for dose estimation. Children’s body weight data were adopted from the new growth charts suggested and developed for Taiwanese children and adolescents [22]. The dust and soil ingestion fits a lognormal distribution, as stated by Özkaynak et al. [21]; the concentrations of insecticides from our previous study were determined to be distributed lognormally by statistical tests. Each of the body weight distributions for different ages on the growth charts appears to be skewed to the right, suggesting a lognormal distribution.

**Figure 1:**
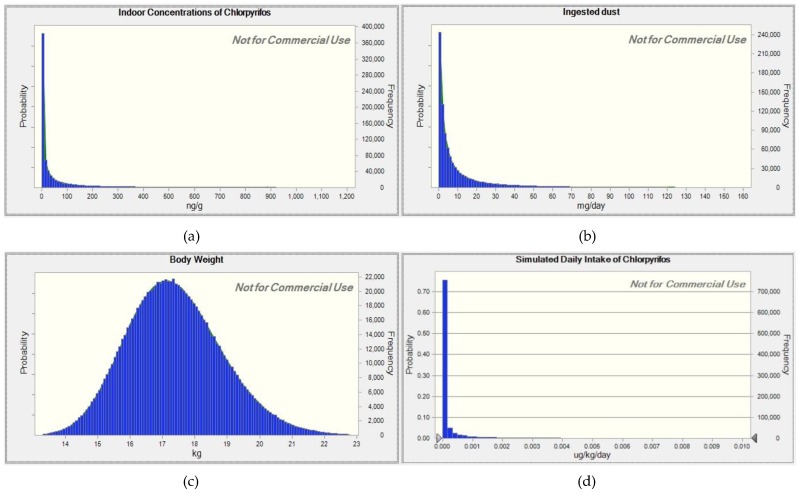
Distributions of variables (**a**–**c**), and result (**d**) for estimation of daily intake of chlorpyrifos.

We applied Monte Carlo simulation as probabilistic modeling using Oracle^®^ Crystal Ball (Fusion Edition, Release 11.1.2.3.000, Oracle^®^ Corp., Redwood Shores, CA, USA), an add-on software to Microsoft^®^ Excel 2010 (Microdoft^®^ Corp., Redmond, WA, USA). The software allows users to set up a distribution of data, instead of a value of mean or median, as input of a variable. At each trial, data are randomly selected following the given distributions of variables to compute an outcome value; such a trial can be repeated up to a hundred million times to derive cumulative outcome values, which form a distribution as well with full simulated statistical information (e.g., mean, median, standard deviation) (Figure 1). For estimation of the daily intakes in this study, the variables of IR, C and BW were set as lognormal distributions with parameters listed in Table 1, and doses of chlorpyrifos and cypermethrin via indoor dust and outdoor soil ingestion were separately derived. Each simulation for dose estimation was performed with 1,000,000 iterations, and 30 replicates were completed to get better estimates.

For risk assessment of children’s exposure to both insecticides, we followed a previous study that expressed the estimated daily intakes as percentages of the Acceptable Daily Dose (ADI) values for chlorpyrifos and cypermethrin [23], which were 0.01 and 0.02 mg/kg/day, in accordance with World Health Organization (WHO) [24].

## 3. Results

The simulation results of daily intakes of chlorpyrifos and cypermethrin via ingestion of indoor dust and outdoor soils for 3–6 year old children are presented in Table 2. As all variables in the model were set to be lognormal distributions, the simulation data were obviously accumulated to form a lognormal distribution, which was confirmed by the fit-probability-distribution feature of the software (Figure 1d). At the 90th percentile of the daily intake, neither insecticide resulted in more than 0.1% of the respective ADI, indicating that the daily intake of chlorpyrifos or cypermethrin for young children in the residential environment was negligible with a probability more than 90%. The percentage of ADI increased as the percentile went up; at the P99, the total daily intake of chlorpyrifos was raised to 1.30% of ADI (exposure to indoor dust and outdoor soils), whereas that of cypermethrin was approximately 2.35% of ADI. In the extreme case of P99.9, the total intakes leaped to 13.1% and 20.0% for chlorpyrifos and cypermethrin, respectively.

Monte Carlo simulation also included sensitivity analysis of modeling, determining which variables have the greatest impact on the model. For the modeling of indoor dust ingestion, the variable of C (concentration of either insecticide) was the one with the greatest impact, bearing a value of contribution to variance around 82%, whereas IR (ingestion rate) only resulted in approximately 18% and BW (body weight) has nearly zero impact (data not shown in table). Ingestion of outdoor soils demonstrated a similar pattern with 91.4% and 8.5% for C and IR, respectively, and no effect for BW. The sensitivity analysis indicated that concentration of insecticide (C) was the principal factor that determines the daily intake.

## 4. Discussion

As the sensitivity analysis showed, the estimated daily intake was mostly determined by the variable of insecticide concentration in dust or soils (C), data of which were distributed unevenly with the majority at the low end but few with extremely high values. The second influential variable, ingestion rate (IR), appeared to be less dispersed than C with a relative standard deviation (RSD = SD/mean) of 1.37 (=36.54/26.65), compared to an RSD of 6.64 (=15.41/2.32) for C of chlorpyrifos. The least variable with impact was body weight (BW), which resulted in an RSD of 0.086, calculated from simulation modeling. It was apparent that variables with large variability turned out to be the determinant factors of simulation models, which was C in this modeling of daily intake.

The simulation modeling indicates that the daily intake of chlorpyrifos or cypermethrin was minimal for the most occasions, suggesting that pesticide contamination in Taiwan’s home environments may not need to worry. The adopted concentration profiles from our previous study [2], however, demonstrate extremely high values as maxima, which rarely but actually have occurred in few of the homes. These high concentrations (e.g., 112.34 µg/g for chlorpyrifos and 134.60 µg/g for cypermethrin in indoor dust) (Table 1), perhaps measured short after an indoor insecticide application, may result in the daily intakes several times higher than the recommended ADIs. Despite the shocking fact, the non-dietary ingestion exposure to insecticides is commonly low as long as care is taken to prevent extreme events from occurring.

Even at P99 or P99.9, the estimated daily intake of chlorpyrifos or cypermethrin is considered safe, because the predicted results (<20% of ADI) are way below the ADIs suggested by WHO [24]. Nevertheless, let us not forget that, other than the non-dietary ingestion route, dietary ingestion of vegetables or agricultural produce is a major route of insecticide exposure. A Chinese study analyzing 2083 vegetables for chlorpyrifos and cypermethrin in Zhejiang Province has completed a risk assessment for daily intakes of both insecticides via dietary ingestion on a probabilistic approach [23], which provides valuable data for comparison with ours (Table 3). Because those analyzed vegetables are also commonly consumed in Taiwan and the regulations for pesticide residues are similar, we assume that the data of risk assessment could be used as replacement of dietary ingestion exposure to insecticides in Taiwan. As seen in Table 3, with a 99% probability the dietary ingestion exposure to either insecticide outweighed the non-dietary ingestion exposure by a large margin; at P99.9. However, the ratio of dietary to non-dietary ingestion exposure shrunk to 3.06 for chlorpyrifos and 1.30 for cypermethrin, suggesting that home environments highly contaminated with insecticides may contribute considerable doses to the daily intakes for young children.

The comparison in Table 3 also indicates that the daily intake via dietary ingestion of either insecticide rises gradually as the percentile goes up, unlike that via non-dietary ingestion remaining at very low levels until reaching the highest percentile. As discussed before, the estimation of daily intake is mostly determined by insecticide concentration; thus, the dissimilarity between the two ingestion routes was probably due to the difference in variability for insecticide residues in vegetables and in dust or soils. The maximum concentrations of either insecticide in vegetables are <10 µg/g, reported by Yuan et al. [23], whereas the environmental data used in this study showed the highest levels above 100 µg/g (Table 1). The low variability for vegetables was probably due to regulations for insecticide applications on vegetable farming, which limited residues of insecticides and narrowed the distribution range. In contrast, concentrations of insecticides in dust may range from none to extremely high levels, as shown by the previous studies that sampled few days after an indoor application of insecticides [3,19]. Even though the ingestion rate of dust was minimal compared to that of vegetables, the estimated daily intake of dust/soil ingestion could be as significant as that of vegetable ingestion in a home environment that is highly contaminated with insecticides.

The trend that dietary ingestion is the major route of insecticide exposure most of the time until non-dietary ingestion becomes significant at the higher percentiles is in support of the work of Xue et al. [25], which used EPA’s SHEDS-multimedia model to estimate pyrethroid intakes for 3–5 years old. They estimated the doses for the general population and specifically for people with pyrethroid use in the homes (i.e., residential use population), and found that exposure via non-dietary ingestion prevailed over that via dietary ingestion above the 95th and 75th percentiles for the general population and residential use population, respectively. The difference between our result and theirs is that the estimated daily intakes of non-dietary ingestion in this study do not top that of dietary ingestion of vegetables at any chance, suggesting that the residential concentrations of insecticides in Taiwan may be lower than those measured from American homes. This is supported by a comparison of pyrethroid concentrations in dust among various studies made in our previous study [2], which shows the lowest median (0.37 μg/g) and P75 values (0.83 μg/g) for Taiwan’s home environments. Despite the slight difference between this work and other western studies, the non-dietary ingestion exposure to insecticides could be considerable as the environmental levels of insecticides are elevated. An indoor application of insecticides could simply take the daily intakes via non-dietary ingestion to the top percentiles if not conducted with care. Should an indoor application be necessary, it has to be done with caution to prevent young children from getting excess exposure to insecticides.

The environmental data used herein came from a pilot study, and may not fully reflect the distribution of insecticide residues in Taiwan’s houses. In southern cities and counties of Taiwan, insecticide applications are more frequently conducted for vector control (e.g., dengue fever) than that in the county from which our data originate during the warm and hot months, and the residential insecticide concentrations are expected to be elevated. Therefore, the non-dietary ingestion exposure to insecticides could be of special concern in those homes with 3–6 year old children.

## 5. Conclusions

Concentrations of residential insecticide residues were the major determinant factor of the estimation of daily intake via non-dietary ingestion for young children. The daily intake was rarely of concern for young children in the homes of Taiwan most of the time unless the environment was highly contaminated with insecticides (e.g., after an indoor application). Should such a contamination occur, the daily intake via non-dietary ingestion could be close to that via dietary ingestion. To avoid high contamination in the environment, insecticides must be applied with caution indoors.

## Figures and Tables

**Table 1:** Distributions of variables used in daily intake estimation for ingestion of dust and soils.

Variable	*N*	Mean		SD		P5	P25	P50		P75		P95	Maximum
Ingestion rate of dust (mg/day) ^a^	1000	26.65		36.54		0.66	4.06	10.80		28.72		100.97	901.96
Ingestion rate of soils (mg/day) ^a^	1000	40.96		78.29		0.15	5.26	15.34		44.85		175.60	1367.37
Indoor concentration of chlorpyrifos in dust (µg/g) ^b^	52	2.32		15.41		<LOD	0.08	0.11		0.30		0.91	112.34
Outdoor concentration of chlorpyrifos in soils (µg/g) ^b^	57	4.27		23.10		<LOD	<LOD	<LOD		0.11		16.19	134.60
Indoor concentration of cypermethrin in dust (µg/g) ^b^	52	18.33		55.92		<LOD	0.11	0.37		0.83		105.79	343.27
Outdoor concentration of cypermethrin in soils(µg/g) ^b^	57	4.29		22.32		<LOD	<LOD	<LOD		0.11		16.47	134.20
**Variable**	**P3**		**P15**		**P25**		**P50**		**P75**		**P85**		**P97**
Body weight of 3–6 year old children at (kg) ^c^	13.5		15.1		15.8		17.3		19.0		20.1		22.9

SD, standard deviation; P#, #th percentile; <LOD, under limit of detection; ^a^ Derived from Özkaynak et al. [21]; ingestion rate of dust is a sum of dust via hand-to-mouth and object-to-mouth behaviors; ^b^ Derived from Hung et al. [2]; ^c^ Derived from Chen et al. [22]; distributions between 3 and 6 years old are combined.

**Table 2:** Non-dietary ingestion exposures in terms of percentage of Acceptable Daily Intake for young children.

Percentile	Chlorpyrifos via Indoor Dust	Chlorpyrifos via Outdoor Soils	Cypermethrin via Indoor Dust	Cypermethrin via Outdoor Soils
P50	<0.001% (<0.001%, <0.001%)	<0.001% (<0.001%, <0.001%)	<0.001% (<0.001%, <0.001%)	<0.001% (<0.001%, <0.001%)
P75	0.002% (0.002%, 0.002%)	0.001% (0.001%, 0.001%)	0.004% (0.004%, 0.004%)	<0.001% (<0.001%, <0.001%)
P90	0.019% (0.019%, 0.019%)	0.007% (0.007%, 0.007%)	0.052% (0.052%, 0.053%)	0.005% (0.005%, 0.005%)
P95	0.079% (0.079%, 0.079%)	0.029% (0.029%, 0.029%)	0.208% (0.207%, 0.209%)	0.023% (0.023%, 0.023%)
P97.5	0.254% (0.253%, 0.255%)	0.102% (0.101%, 0.102%)	0.629% (0.627%, 0.631%)	0.080% (0.079%, 0.080%)
P99	0.884% (0.880%, 0.887%)	0.412% (0.410%, 0.414%)	2.042% (2.035%, 2.049%)	0.310% (0.308%, 0.311%)
P99.9	8.313% (8.234%, 8.392%)	4.819% (4.782%, 4.856%)	16.89% (16.75%, 17.04%)	3.155% (3.124%, 3.186%)

Numbers in parenthesis represent the 95% confidence intervals.

**Table 3:** Comparison of non-dietary and dietary ingestion exposures in terms of percentage of ADI.

Percentile	Chlorpyrifos via Non-Dietary Ingestion	Chlorpyrifos via Dietary Ingestion ^a^	Cypermethrin via Non-Dietary Ingestion	Cypermethrin via Dietary Ingestion ^a^
P50	<0.001% (<0.001%, <0.001%)	1.39% (1.35%, 1.42%)	<0.001% (<0.001%, <0.001%)	1.67% (1.64%, 1.70%)
P75	0.003% (0.003%, 0.003%)	NA	0.004% (0.004%, 0.004%)	NA
P90	0.026% (0.026%, 0.026%)	15.52% (15.35%, 15.70%)	0.057% (0.057%, 0.058%)	10.55% (10.44%, 10.67%)
P95	0.108% (0.108%, 0.108%)	NA	0.231% (0.230%, 0.232%)	NA
P97.5	0.356% (0.354%, 0.357%)	24.07% (23.69%, 24.47%)	0.709% (0.706%, 0.711%)	15.94% (15.68%, 16.19%)
P99	1.296% (1.290%, 1.301%)	29.03% (28.43%, 29.66%)	2.352% (2.343%, 2.360%)	19.09% (18.70%, 19.52%)
P99.9	13.10% (13.02%, 13.25%)	40.16% (38.39%, 42.41%)	20.05% (19.87%, 20.23%)	26.07% (24.87%, 27.42%)

NA, not available; Numbers in parenthesis represent the 95% confidence intervals; ^a^ Data for 2–6 years old in the whole province of Zhejiang, derived from Yuan et al. [23].
